# ProteinSeq: High-Performance Proteomic Analyses by Proximity Ligation and Next Generation Sequencing

**DOI:** 10.1371/journal.pone.0025583

**Published:** 2011-09-29

**Authors:** Spyros Darmanis, Rachel Yuan Nong, Johan Vänelid, Agneta Siegbahn, Olle Ericsson, Simon Fredriksson, Christofer Bäcklin, Marta Gut, Simon Heath, Ivo Glynne Gut, Lars Wallentin, Mats G. Gustafsson, Masood Kamali-Moghaddam, Ulf Landegren

**Affiliations:** 1 Department of Immunology Genetics and Pathology, Uppsala University, Uppsala, Sweden; 2 Department of Medical Sciences, Clinical Chemistry, University Hospital, Uppsala, Sweden; 3 Halo Genomics AB, Uppsala Sweden; 4 Olink Biosciences, Uppsala, Sweden; 5 Cancer Pharmacology and Informatics, Department of Medical Sciences, Uppsala University, Uppsala, Sweden; 6 Centro Nacional de Análisis Genómico, Barcelona, Spain; 7 Science for Life Laboratory, Uppsala, Sweden; Stanford, United States of America

## Abstract

Despite intense interest, methods that provide enhanced sensitivity and specificity in parallel measurements of candidate protein biomarkers in numerous samples have been lacking. We present herein a multiplex proximity ligation assay with readout via realtime PCR or DNA sequencing (ProteinSeq). We demonstrate improved sensitivity over conventional sandwich assays for simultaneous analysis of sets of 35 proteins in 5 µl of blood plasma. Importantly, we observe a minimal tendency to increased background with multiplexing, compared to a sandwich assay, suggesting that higher levels of multiplexing are possible. We used ProteinSeq to analyze proteins in plasma samples from cardiovascular disease (CVD) patient cohorts and matched controls. Three proteins, namely P-selectin, Cystatin-B and Kallikrein-6, were identified as putative diagnostic biomarkers for CVD. The latter two have not been previously reported in the literature and their potential roles must be validated in larger patient cohorts. We conclude that ProteinSeq is promising for screening large numbers of proteins and samples while the technology can provide a much-needed platform for validation of diagnostic markers in biobank samples and in clinical use.

## Introduction

Recent years have brought the opportunity to comprehensively analyze genomes, epigenomes and transcriptomes through next generation sequencing (NGS) [1]. However, analogous methods have been lacking to measure large sets of proteins in biological samples, while so far the search for clinically useful protein biomarkers has met with limited success [2], [3]. Greatly improved analytical methods are therefore required in basic research and to validate protein biomarkers by characterizing their distribution in large series of patient samples, meeting stringent requirements for sensitivity and specificity and with minimal consumption of precious sample material. The aim is to find markers that can help diagnose disease, preferably at early stages, and diagnose responsiveness to specific therapies, or identify signs of relapse [4].

A number of approaches have been presented for protein detection and quantitation in high-throughput. Mass-spectrometry (MS) is combined with specific affinity reagents in methods such as stable isotope standards and capture by anti-peptide antibodies (SISCAPA) [5], [6] and with multiple reaction monitoring (MRM) [7]. Such assays demonstrate improved sensitivity over other MS-based approaches but sample preparation is relatively time-consuming and costly, and the degree of multiplexing is currently limited.

Arrays of antibodies or other binding agents can be used to capture proteins from biological samples for subsequent measurement in assays that can be scaled to large numbers of analytes [8], [9], [10]. A recent, interesting variant of this approach employs large sets of high-affinity DNA aptamers for parallel capture of proteins in biological samples, followed by detection and quantitation [11], [12].

Traditional sandwich immunoassays, which rely on dual-recognition of target proteins by pairs of antibodies for capture and signal reporting, offer an additional level of specificity over single-binder assays. They can therefore in general provide improved sensitivity of detection in complex biological specimens. Such methods are robust and easy to use, and variants where multiple proteins are detected in parallel are commercially available. However, as the number of proteins investigated in the same assay increases, risks for detection by non-cognate antibody pairs grows rapidly, gradually undermining the added specificity by dual recognition [13], [14].

The proximity ligation assay (PLA) [15] is an immunoassay where pairs of oligonucleotide-labeled antibodies - PLA probes - are employed to detect an antigen of interest. When two PLA probes bind the same antigen, the attached oligonucleotides are brought in proximity, allowing these to be ligated upon addition of a short complementary oligonucleotide. The ligated DNA strands serve as reporter molecules that can be readily detected using nucleic acid analysis techniques such as quantitative real-time PCR (qPCR). The ligation and amplification steps provide opportunities to constrain detection reactions so that only cognate pairs of antibodies can give rise to detectable signals, thereby avoiding cross-reactions. This property renders the assays highly suitable for multiplexing. So far, multiplex solution-phase PLA tests have been described for sets of 24 antigens simultaneously [16], [17], [18].

We recently developed a solid-phase version of PLA (SP-PLA). In this assay, an antibody immobilized on a solid support acts as a capture reagent for localized enrichment of the antigen from a complex mixture of proteins, such as serum or plasma [19]. After washes, a pair of PLA probes is added followed by washes and ligation of oligonucleotides brought in proximity. The solid support allows washes to remove excess PLA probes and other molecules that could interfere with detection of the protein of interest. The unique requirement that each targeted protein is recognized via three binding events in order to be recorded provides exceptional specificity of detection compared to single-binder or sandwich assays. This, in combination with the use of PCR amplification to detect signals, allows for high specificity and sensitivity of detection, and a broad dynamic range for protein quantification.

Herein we report the development of multiplexed solid-phase PLA coupled with NGS to digitally record patterns of protein abundance in a method we call ProteinSeq. We demonstrate simultaneous detection of 36 protein analytes, including one internal control, in only 5 µl blood plasma samples. The digital nature of NGS coupled with an exponentially increasing sequencing capacity and rapidly decreasing sequencing cost renders ProteinSeq promising for high-performance and high-throughput biomarker validation in large clinical studies.

We used ProteinSeq to digitally record protein levels in plasma samples from patients with CVD compared to normal controls, revealing potentially important protein biomarkers.

## Results

**Figure 1:**
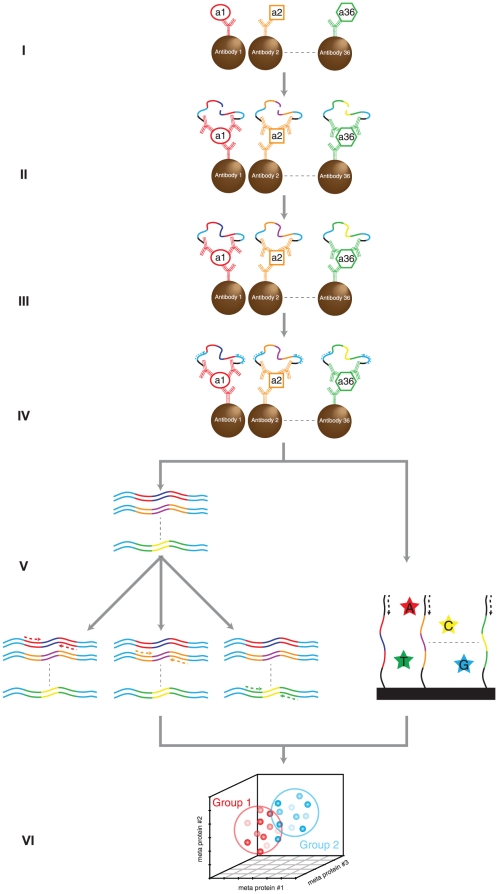
Principle of multiplex SP-PLA. Pools of microparticles, each coated with one of the antibodies, are mixed with the sample (I). After washes, pairs of PLA probes directed against each of the proteins are incubated with the microparticles (II), followed by washes and ligation of the attached DNA strands (III). Ligated molecules are first amplified with primers directed against sequences present on all ligated DNA molecules (IV). This universal pre-amplification is then followed by preparation of the reporter strands for next generation sequencing, or for qPCR (V). Subsequently the abundance of each protein is calculated and multivariable classification of cases and controls is performed (VI).

We have developed a scalable approach for sensitive, parallel protein detection using minimal sample aliquots (**Fig. 1**). We first established basic characteristics of the assay, before proceeding to investigate plasma protein changes in samples from patients with CVD.

A first requirement for high-throughput protein assays is the ready access to suitable sets of reagents. Herein we used single affinity-purified polyclonal antibody preparations, raised against the whole or a large portion of the targeted proteins, as a source of all three affinity reagents required for detection of any protein by SP-PLA. Aliquots of the antibodies were immobilized on paramagnetic microparticles or modified by covalent attachment of oligonucleotides via their 5′ or 3′ ends. A rapid and convenient protocol was developed for construction of up to 48 pairs of antibody-oligonucleotide conjugates per 96-well plate within three hours. The success rate of obtaining reagents for sensitive protein detection using a commercial source of antibodies was a highly satisfactory 90%, permitting construction of large reagent repertoires.

**Figure 2:**
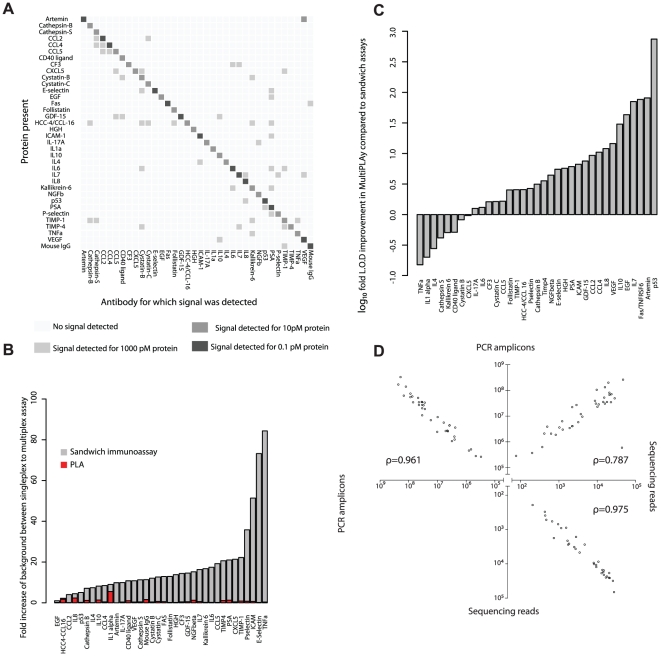
a) Cross-reactivity table. The figure reflects the signals obtained when each protein in the panel was incubated individually with all PLA probes of the panel. Correct signals are indicated along the diagonal. The colors indicate the protein concentration required to elicit detection signals significantly exceeding the background. Recombinant proteins at known concentrations were used for the purpose of this experiment. **b**) **Change in background signals as a result of multiplexing**. The background signals were measured for every antibody when incubated individually or in combination with all other antibodies of the panel for multiplex SP-PLA and sandwich immunoassays. The y-axis shows the increase of background when moving from individual to multiplex reactions. **c**) **Limit of detection comparison between multiplex SP-PLA and sandwich immunoassay**. The log_10_ fold change in LOD between multiplex SP-PLA and sandwich immunoassay. **d**) **Correlation between results of sequencing and qPCR**. Correlation between two replicated measurements of the same blood sample by qPCR (upper left), by sequencing (lower right), and between measurement by realtime PCR and sequencing (upper right). The axes represent numbers of starting DNA amplicons for PCR measurements and normalized numbers of reads for sequencing.

To evaluate the specificity of multiplex SP-PLA, we first assessed the tendency by each of the 36 different antibody sets to detect any of the 36 targeted antigens in the panel. Individual dilutions for every protein in the panel were prepared using recombinant proteins and each one of the target proteins was analyzed at high, medium or low concentrations (1 nM, 10 pM and 0.1 pM, respectively in a total volume of 45 ul) using qPCR for readout of PLA reaction products (**Fig. 2a**). For a conversion of molar concentrations of each protein to protein concentrations in pg/µl, please refer to **Table S1**. Unspecific signals were classified as significant if they exceeded background levels by at least two standard deviations. All but one of the instances where unspecific signals were observed concerned proteins present at the high concentration of 1 nM, a level substantially higher than the blood level for these markers in both healthy and diseased individuals. Furthermore, since the unspecific signals are not reciprocal, i.e. the opposite combinations of reagents and target molecules did not elicit unspecific signals, any contribution to detection signals by cross-reactivity between analytes can be estimated and accounted for.

In order to avoid risks of detection reactions involving non-cognate PLA probe pairs, that is antibodies that are not expected to bind the same protein, the assays were designed so that only correct pairs of oligonucleotide-modified antibodies could be joined by ligation (see **Methods and Fig. S1**). We thus expected that background signals would show a lesser tendency to increase as more sets of antibodies are used compared to the situation for sandwich immunoassays. To investigate this we measured background signals in individual PLA tests compared to multiplex PLA, and we used the same polyclonal antibodies in conventional sandwich immunoassays performed singly or in multiplex in a bead-based system (Luminex). The results, summarized in **Figure 2b** establish that background signals from PLA do not increase significantly when going from individual to multiplex tests. In contrast, using the conventional sandwich immunoassay design background signals increased greatly but variably as the number of detection reagents was increased, as has been noted by others [20], [21], [22], [23]. Accordingly PLA is promising for higher degrees of multiplexing than what can be achieved using conventional sandwich immunoassays.

Next we compared multiplex SP-PLA with qPCR readout and sandwich immunoassays with respect to assay sensitivity (lower limit of detection, LOD) and the concentration range over which proteins could be quantified (dynamic range) using the same antibody preparations. Multiplex SP-PLA exhibited an average overall improved sensitivity of 40-fold compared to sandwich assays (**Fig. 2c** **and Figure S2**). Among 35 proteins, multiplex SP-PLA had lower LOD for 27 analytes, while the sandwich immunoassay was slightly more sensitive for the remaining eight, perhaps because of a limited epitope range for these antisera. Multiplex SP-PLA had a median dynamic range of five orders of magnitude for the 35 targeted proteins, compared to three orders of magnitude for the sandwich immunoassays. A list of detection limits for each antigen for both multiplex SP-PLA and sandwich assays can be found in **Table S2**.

We specifically evaluated the correlation between data obtained by multiplex SP-PLA with qPCR readout and sandwich immunoassays on a set of 46 plasma samples for six proteins, namely E-selectin/CD62E, Fas-Ligand/TNFSF6, CCL16/HCC-4, CXCL8/IL8, GDF-15 and Kallikrein-3/PSA. The median Pearson correlation coefficient in these experiments was 0.70 (min = 0.52, max = 0.87) across all samples. The levels of all 35 proteins investigated by multiplex SP-PLA on two separate occasions in 45 plasma samples exhibited a median Pearson correlation coefficient of 0.75 (min = 0.05, max = 0.96).

The measurement of proteins via multiplex SP-PLA and qPCR provides an analogue record of the protein complement in the investigated samples. By contrast, NGS offers a means to digitally record PLA-products that represent protein profiles in blood samples to ultimately improve precision and sample throughput by a procedure we call ProteinSeq.

To optimize detection of proteins over broad ranges of abundance and avoid overrepresentation of PLA products representing more abundant proteins we found that we could adjust the detection efficiency. For proteins expressed at high levels we could predictably lower the reporting rate by decreasing the concentration of the corresponding PLA probes (**see Methods and Table S3**).

We developed a protocol that significantly simplified preparation of sequencing libraries for PLA ligation products compared to commercially available procedures (**Fig. S3**). Using this protocol, amplified ligation products from different patient samples were labeled with a set of twelve indexes, allowing us to analyze 96 patient or control samples in the eight lanes of an Illumina Genome Analyzer IIx.

We applied ProteinSeq to investigate biobank plasma samples from a group of CVD patients (n = 63) and matched healthy controls (n = 19). Each sequenced product was assigned to a plasma sample using the index sequences. Of the total reads, 92% contained a recognizable index, allowing up to one mismatch. The identity of the detected protein was inferred from two specific tag sequences contributed by the two oligonucleotides used in the PLA probes. From those reads that contained a recognizable index, 77.9% mapped uniquely to a reference sequence. The reference sequence contained all possible ligation products and not just the expected pairs of sequence tags, while allowing for up to five mismatches. Out of all the uniquely mapped reads, as little as 1.5% involved incorrect probe pairs (**see Fig. S4**). This demonstrates that the constrained ligation efficiently suppresses unspecific signals originating from recognition of proteins by non-cognate pairs of antibodies, supporting the notion that further multiplexing should be possible.

The quantitative precision of measurement by sequencing was marginally better than for qPCR (CV 22% vs. 29% averaged for all proteins). In addition, we used data obtained from one patient sample for which we acquired replicate measurements to establish that correlation between replicate measurements for sequencing and qPCR was similar for the two methods (**Fig. 2d**).

**Figure 3:**
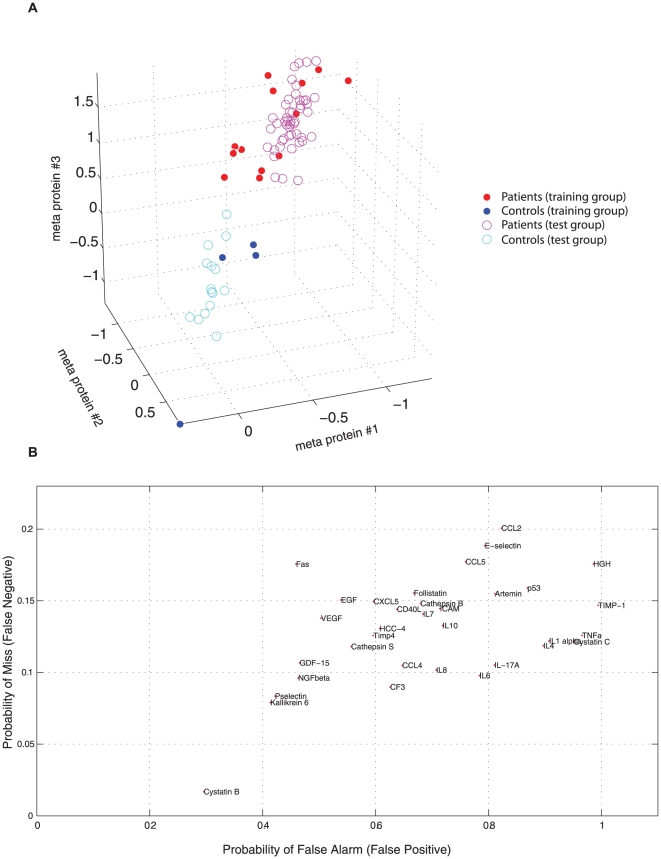
a) Supervised multivariate classification. Typical results of a multivariate classifier design using 80% of the CVD samples. The cyan (control) and magenta (patient) open circles indicate the positions of the training examples in the compressed three-dimensional meta-protein space, created by the supervised procedure to classify the samples (see methods). The blue (controls) and red (patients) filled circles indicate the positions of the external test examples used to evaluate the particular classifier designed. These examples were assigned to the most common class among the three closest training examples (3-nearest neighbour classification). **b**) **Supervised univariate analysis of individual proteins**. Results obtained from the CVD samples by a supervised univariate analysis of the individual proteins with respect to their discriminatory power when they were used individually for 3-nearest neighbour classification. Each protein is displayed in terms of estimates of the probabilities of false alarm and miss that one would obtain if an optimal cut-off level for that particular protein was used alone to distinguish patients and controls.

A multivariate classification method, introduced here for the first time, was used for the analysis of the multidimensional data generated by ProteinSeq. Prior to analysis two patient samples containing missing values were removed from the original set of 63 patient samples, resulting in a total of 61 patient and 19 control samples. The classifications resulted in an average false positive rate estimate of 19% and an average negative rate estimate of 4% for the novel projection method. These estimates are based on 5000 different designs using 48 patient examples plus 15 control examples (80% of all the data) each time. Each design was then tested using an external set of test examples consisting of 13 patients and 4 controls, from which the estimates were calculated. Figure 3a illustrates the result of one of the 5000 different classifier designs performed and evaluated. These findings were validated by two commonly employed classification methods, namely nearest shrunken centroid (NSC) and random forest (RF). For further details on the novel projection method as well as the comparison with the two established classification methods please refer to the Methods and Material S1.

To identify the most promising potential biomarkers among proteins included in our panel, we performed a 3-nearest neighbor classification for each protein individually (**Fig. 3b**). Cystatin-B stood out as the most informative protein for discriminating patients and controls. This protein resulted in a false positive rate of 28% and a false negative rate of only 2%. Both rates were estimated as averages over 1000 repeated external 5-fold cross validations. Kallikrein-6 and P-selectin both came second as informative markers with an estimated false positive rate of approximately 43% and false negative rate of 8%. Of these three proteins, P-selectin has previously been identified as a promising biomarker for CVD [24], [25] but no reports were found on Cystatin-B and Kallikrein-6, motivating further analyses of these biomarkers in larger numbers of samples.

## Discussion

We report herein the development and application of a technology for highly specific and parallel protein measurements with readout via DNA sequencing – ProteinSeq – suitable for sensitive validation of biomarker candidates in plasma samples. The use of a solid support along with the requirement for three binding events to generate a detection signal ensures specificity of detection, while low nonspecific ligation and efficient PCR amplification of ligation products contribute to the sensitivity of ProteinSeq analyses. The recognition events per target by three antibodies also provides an opportunity to characterize more challenging targets, such as complexes of interacting proteins or proteins having undergone post-translational modifications [26], [27]. The requirement for target binding by three antibodies could prove disadvantageous if the protein size imposes a limitation of the number of available epitopes. However, we have not observed any correlation between assay performance and protein size for the 36 proteins included in our panel. Indeed, some of the best performing assays in the panel are against relatively small proteins such as interleukin-7 with a mass of approximately 17 kilodalton. The use of polyclonal antibodies, while convenient, fails to ensure that individual proteins are bound by antibodies having the required combination of oligonucleotides. Work is in progress to rapidly characterize optimal trios of clonal affinity reagents (monoclonal antibodies or recombinant binders) for solid-phase PLA tests.

We demonstrate the potential of the method for extensive biobank analyses by simultaneously investigating 35 analytes in 5 µl aliquots of blood plasma with both minimal cross-reactivity and improved limits of detection compared to traditional sandwich immunoassays. This suggests that it will be possible to extend the method for parallel analyses of much larger numbers of proteins. Finally, we illustrate herein for the first time the use of NGS as a readout through ProteinSeq, allowing digital protein measurement in plasma samples.

ProteinSeq allows broad sets of analytes to be analyzed in the same sample without a need to divide analyses in separate panels based on the relative abundance of the target proteins in blood. The use of single polyclonal antibody preparations to construct all three classes of reagents needed for detection of each protein avoids the need to identify suitable combinations of monoclonal antibodies, thus overriding one of the important limitations for analysis of large sets of proteins.

ProteinSeq lends itself for much higher level of multiplexing than conventional sandwich immunoassays. This is supported by the observation that background signals were not significantly different between individual and multiplex assays, suggesting that far greater multiplexing should be possible. By contrast, we observed increased background with multiplexing of a conventional sandwich immunoassay.

We demonstrated the potential of ProteinSeq for biomarker validation in a study involving patients with coronary disease. In the CVD sample set three proteins stood out as promising markers in the univariate analysis, namely Cystatin-B, Kallikrein-6 and P-selectin. P-selectin has been previously suggested as a promising biomarker for CVD [24], [25] but no reports were found on Cystatin-B and Kallikrein-6, indicating that these might represent novel biomarkers. Given that the number of samples used herein is relatively small the potential of Cystatin-B and Kallikrein-6 should be further and thoroughly investigated. Such a study would require analyses of large numbers of samples within a reasonable time frame, whereas for the identification of further biomarkers, large numbers of proteins would have to be analyzed as well.

In this context DNA sequencing allows for specific, sensitive and precise quantitation of ProteinSeq ligation products, providing very high levels of sample and marker throughput. Digital protein expression data can greatly augment analytical performance, and the cost for such analyses will diminish rapidly, as the cost of DNA sequencing is decreasing at a very rapid rate [28]. By optimally encoding the identities of proteins and samples, only a small number of nucleotides need be sequenced for unambiguous identification and as shown herein little sample preparation is required before PLA products are sequenced. We also demonstrate, that reporting of proteins from different abundance classes can be conveniently compressed with minimal loss of precision to avoid excessive sampling of products for abundant proteins.

In conclusion, ProteinSeq provides opportunities for highly multiplexed assays of proteins or protein variants in minimal sample aliquots. It can thus allow validation of protein expression patterns in biobank samples and in prospective studies, and the method can provide a platform for clinical use.

## Materials and Methods

### Recombinant proteins and antibodies

All recombinant human proteins and all antibodies, with the exception of IgG from mouse serum, were from R&D Systems. The IgG from mouse serum was purchased from Sigma-Aldrich. Both unbiotinylated and biotinylated affinity-purified forms of the antibodies were used. All antibodies were produced in goats with the exception of ICAM-1/CD54, E-selectin/CD62E and P-selectin/CD62P that were produced in sheep. A list of all antibodies can be found in **Table S4.**

### Oligonucleotides

Oligonucleotides for conjugation to antibodies for purposes of producing PLA probes were equipped with a thiol group at the end to be attached to the antibody. PLA oligonucleotides with a 5′ thiol modification were purchased from IDT while oligonucleotides with a 3′ thiol modification were obtained from Eurogentec. All protein-specific and universal primers as well as the ligation template were from IDT. The ligation template was designed to allow specific ligation of all 36 oligonucleotide pairs. This was achieved by designing the oligonucleotides on PLA probe pairs so that each pair formed a nick at a unique position when hybridizing to the ligation template. This sliding splint-mechanism served to ensure that only cognate pairs of oligonucleotides, attached to antibodies could be joined by ligation. The sliding splint concept is described in greater detail in **Figure S1**. All oligonucleotides used for library preparation prior to sequencing were from IDT. All oligonucleotides were HPLC purified. For a complete list of all oligonucleotides refer to **Tables S5, S6, S7, S8**.

### Solid support preparation

The immobilization of biotinylated capture antibodies on Dynabeads MyOne Streptavidin T1 (Invitrogen) paramagnetic microparticles and the subsequent treatment and storage of the beads was performed as described previously [19].

### PLA probes preparation

Conjugation of oligonucleotides to antibodies was performed as described by Soderberg *et al.* [29] with minor modifications. The buffer used for the reduction of oligonucleotides was 1xPBS, pH 7.4 containing 5 mM EDTA. Upon conjugation, the reactions were purified in 96-well filter plates (MultiScreenHTS-HV Plate, Millipore) to which 500 µl lllustra Sephadex™ G-50 DNA Grade resin (GE Healthcare) were added.

### CVD sample set

The CVD sample set comprised three patient subgroups. For each of the subgroups, sample collection was performed as follows: *Group 1*: within 72 h after onset of unstable angina or minor myocardial infarction (n = 16); *Group 2*: upon arrival at the hospital with sudden total thrombotic occlusion of a coronary artery as evidenced by electrocardiographic ST segment elevation, motivating reperfusion therapy (n = 23); *Group 3*: in the emergency department for patients with chest pain due to suspicion of unstable coronary artery disease or myocardial infarction (n = 24). Samples were stored at −70°C until analyzed. The control samples were collected from age- and gender-matched individuals with no signs or symptoms of coronary disease (n = 19). Descriptive statistics of the patient and control groups can be found in **Table S9**. All participants provided written informed consent and all studies had ethical committee approvals as stated in the original publications [30], [31], [32], [33].

### Solid-phase multiplex PLA

For each reaction we used 0.1 µl of microbeads modified by the addition of the biotinylated capture antibody, corresponding to 0.75 ng of capture antibody, for every analyte in our multiplex panel. After combining beads individually modified with antibodies against all 36 analytes, the storage buffer was removed and beads were re-suspended in PLA buffer (1 mM D-biotin (Invitrogen), 0.1% purified BSA (New England Biolabs), 0.05% Tween 20 (Sigma-Aldrich), 100 nM goat IgG (Sigma-Aldrich), 0.1 g/l salmon sperm DNA (Invitrogen), 5 mM EDTA, PBS). We used 5 µl of the re-suspended bead mix in each reaction.

Recombinant proteins were serially diluted in PLA buffer in concentrations ranging from 1 nM to 10 fM and with one negative control to determine background noise.

Upon mixture of 5 µl bead mix and 45 µl of diluted recombinant protein or ten-fold diluted plasma sample from patients and controls, the reactions were incubated for 1.5 hours at room temperature (RT) with constant rotation. This was followed by two washes using wash buffer (PBS, 0.05% Tween 20) while the reaction wells were positioned on a 96-well plate magnet (Perkin Elmer Life Sciences).

After washes, 50 µl of PLA probe mix with each oligonucleotide-conjugated antibody for a final concentration of 0.5 nM was added to each reaction well. Reactions were incubated for 1.5 h at RT with constant rotation, followed by two washes as described above.

Next, 50 µl of ligation mix was added (1X AmpLigase buffer (Epicentre biotechnology), 0.5 mM NAD (Sigma), 0.1 U AmpLigase (Epicentre biotechnology), 100 nM ligation splint). After 10 min at 50°C, the beads were washed once.

Subsequently 50 µl of universal PCR mix (1X PCR buffer (Invitrogen), 3 mM MgCl_2_ (Invitrogen), 100 nM universal forward and reverse primers, 1.5 U Platinum Taq DNA polymerase (Invitrogen), 0.2 mM dNTP mix (dATP, dGTP, dCTP, dUTP) (Fermentas), 0.1 U uracil-N-glycosylase (Fermentas)) was added to every well and pre-amplification was carried out using the following thermocycling protocol, 10 min at 95°C, followed by 15 cycles of 15 sec at 95°C, 1 min at 62°C, and 1 min at 72°C.

Separate qPCRs were carried out for each ligation product in 384-well plates (Applied Biosystems) in which 2 µl of 0.5 µM specific primer pairs for individual ligation products had been previously added using a Hydra dispenser (Robbins Scientific). Universally amplified products were diluted fifty-fold in PCR mix and 8 µl were added to the primer-containing 384-well plates. The qPCR thermocycling protocol included an initial incubation at 95°C for 2 min followed by 40 cycles of 15 sec at 90°C and 1 min at 60°C.

### Sandwich immunoassays

Aliquots of the same polyclonal antibodies used for PLA were also coupled to Magnetic MAGPLEX microspheres (Luminex Corporation; identities 7–9, 12–15, 18–22, 25–30, 33–39, 42–48, 65–68) according to the manufacturer's recommended protocol.

For each assay we used 750–1000 beads suspended in 5 µl of assay buffer (1xPBS, 1% BSA). All blood samples were diluted ten-fold in assay buffer prior to analysis for a final reaction volume of each sample of 45 µl. Samples were incubated with the beads for 1.5 h in darkness with shaking at 650 rpm, followed by washing with PBS-T (1xPBS, 0.05% Tween 20). Next, 50 µl of detection solution was added to the beads, comprising all biotinylated antibodies, each at a concentration of 3.33 nM. Upon washing, 50 µl of 0.5 µg/ml streptavidin-coupled R-phycoerythrin (Invitrogen Ltd) were added to the reaction and incubated for 30 min. After removing the solution 125 µl PBS-T was added and the reactions were transferred to a Luminex Lx200 instrument for further analysis.

### Library preparation for sequencing

ProteinSeq reactions were performed by adjusting the concentration of certain PLA probes as shown in **Table S3**. This was done to ensure that all resulting ligation products would be within the same dynamic range of three orders of magnitude.

Universal PCR amplification was performed in 50 µl special PCR buffer (50 mM KAc, 20 mM Tris-HAc, 3 mM MgAc_2_ pH 7.6), 100 nM universal forward and universal reverse primers, 1.5 U Platinum Taq DNA polymerase, 0.2 mM dNTP mix (Fermentas), 0.1 U uracil-N-glycosylase (Fermentas)) with the thermal profile described above and for 35 cycles.

Sample indexes were introduced by mixing 24 µl of each pre-amplified reaction with 26 µl of indexing mix (0.5 µM PCR Index forward and reverse primers, special PCR buffer with 1.5 U Platinum Taq DNA polymerase and 0.2 mM dNTP mix (Fermentas)) and incubating at 95°C for 10 min, 95°C for 15 sec, 62°C for 1 min and 72°C for 1 min.

Ten µl of each product were subsequently amplified with 40 µl enrichment mix (Special PCR buffer, 0.5 µM enrich forward and reverse primer, 1.5 U Platinum Taq DNA polymerase and 0.2 mM dNTP mix) by incubating at 95°C for 10 min and subsequently performing 3 cycles of 95°C for 15 sec, 65°C for 30 sec and 72°C for 30 sec.

All reactions were purified in GFX™ columns (GE Healthcare) followed by size selection for 199 bp on 2% size-selection E-gel (Invitrogen) using 50 bp DNA size ladder (Molecular Probes). Selected products were quantified by qPCR and combined to 7 groups each containing 12 indexes prior to sequencing.

### Sequencing

Sequencing was carried out using an Illumina Genome Analyzer IIx sequencer. Pools of libraries were introduced into the eight channels of a single-end flow cell. 95 bases of sequence were prepared.

### Sequencing data analysis

All reads were initially de-multiplexed by using the 6 bp sample index sequence, allowing for a maximum of one mismatch. De-multiplexed reads were then aligned to the reference sequence using the GEM aligner to perform an exhaustive mapping allowing for a maximum of five mismatches. Only uniquely mapping reads were used for subsequent analyses, where a read was declared unique with *n* mismatches if it did not match with less than *n* mismatches, matched once with exactly *n* mismatches, and did not match with *n*+1 mismatches.

The total number of reads obtained for every DNA molecule that corresponded to each protein included in the ProteinSeq panel was used for the analysis. Prior to analysis the absolute number of sequencing reads was normalized against the internal PLA control (mouse IgG). For a more detailed description of sequencing statistics please refer to **Figure S4**.

### Multivariate data analysis

All the multivariate data analysis was performed using in-house code written in Matlab (Mathworks, USA) and code written in R [34]. Codes are available upon request. For details regarding missing values (imputation), the three different supervised learning methods employed, the performed standardization of the protein levels to a common scale and the careful performance estimation, see **Material S1**.

## Supporting Information

Figure S1 **The concept of the sliding splint design.** Each multiplex SP-PLA oligonucleotide pair is ligated on the sliding splint with a single nucleotide shift relative to the previous one. In that way incorrect oligonucleotide combinations will not form proper substrates for ligation and cannot be ligated.
(EPS)

Figure S2 **Limit of detection comparison between multiplex SP-PLA and sandwich immunoassay.** The LOD of multiplex SP-PLA and sandwich immunoassay for every protein in the panel. The LOD is presented in
(EPS)

Figure S3 **Library preparation for sequencing.** Sample barcodes are introduced in the pre-amplified ligation products by polymerization. Subsequently, barcoded samples are mixed in one tube prior to be sequenced. For more information please refer to the **Methods** section.
(EPS)

Figure S4 **Sequencing statistics.** Columns indicate the absolute number of reads for each category in the x-axis. The number of reads of each category is also shown as a percentage of the total number of reads.
(EPS)

Table S1 **Conversion of molar concentrations to concentrations in pg/μl.** The highest molar concentration (pM) for every protein used in the cross-reactivity experiment is converted to mass per volume concentration (pg/μl). In addition reaction volumes are provided.
(DOCX)

Table S2 **Detection limits for each protein included in the panel.** The limits of detection for each protein included in the panel for both multiplex SP-PLA and sandwich immunoassays are presented in pg/ml.
(DOCX)

Table S3 **Adjusted concentration of each PLA probe.** Probe concentrations were adjusted in order to decrease PLA reporting efficiency and thus limit the dynamic range of different ligation products.
(DOCX)

Table S4 **List of antibodies used as PLA probes.**
(DOCX)

Table S5 **Sequences of PLA arms.** Sequences of all oligonucleotides conjugated on antibodies by their 5′, which was modified by addition of a thiol group.
(DOCX)

Table S6 **Sequences of PLA arms.** Sequences of all oligonucleotides conjugated on antibodies by their 3′, which was modified by addition of a thiol group.
(DOCX)

Table S7 **PCR primer sequences.** Sequences of all PCR primers used in multiplex SP-PLA with qPCR readout.
(DOCX)

Table S8 **Library preparation oligonucleotide sequences.** Sequences of all oligonucleotides used for the preparation of sequencing libraries.
(DOCX)

Table S9 **Characteristics of patient and control groups.**
(DOCX)

Material S1 **Supplementary Material.**
(DOCX)
