# Supplementary material for: ProteinSeq: High-Performance Proteomic Analyses by Proximity Ligation and Next Generation Sequencing
Source: PLoS One. 2011 Sep 29;6(9):e25583. doi: 10.1371/journal.pone.0025583 (PMC3183061; doi:10.1371/journal.pone.0025583)
Supplement: Table S1 — Conversion of molar concentrations to concentrations in pg/μl. The highest molar concentration (pM) for every protein used in the cross-reactivity experiment is converted to mass per volume concentration (pg/μl). In addition reaction volumes are provided. (DOCX) [file pone.0025583.s005.docx]

| **Protein name** | **Concentration (pM)** | **Concentration (pg/μl)** | **Volume (μl)** |
| --- | --- | --- | --- |
| Artemin | 1000 | 24.0 | 50 |
| Cathepsin B | 1000 | 33.5 | 50 |
| Cathepsin S | 1000 | 33.5 | 50 |
| CCL2 | 1000 | 8.7 | 50 |
| CCL4 | 1000 | 7.5 | 50 |
| CCL5 | 1000 | 7.8 | 50 |
| CD40 ligand | 1000 | 17.0 | 50 |
| CF3 | 1000 | 26.0 | 50 |
| CXCL5 | 1000 | 8.4 | 50 |
| Cystatin B | 1000 | 12.0 | 50 |
| Cystatin C | 1000 | 15.0 | 50 |
| E-selectin | 1000 | 58.6 | 50 |
| EGF | 1000 | 6.0 | 50 |
| Fas/TNFRSF6 | 1000 | 90.0 | 50 |
| Follistatin | 1000 | 31.0 | 50 |
| GDF-15 | 1000 | 26.0 | 50 |
| HCC-4/CCL 16 | 1000 | 11.0 | 50 |
| HGH | 1000 | 22.0 | 50 |
| ICAM | 1000 | 50.0 | 50 |
| IL-17A | 1000 | 18.6 | 50 |
| IL1 alpha | 1000 | 32.0 | 50 |
| IL10 | 1000 | 14.0 | 50 |
| IL4 | 1000 | 20.3 | 50 |
| IL6 | 1000 | 17.0 | 50 |
| IL7 | 1000 | 8.0 | 50 |
| IL8 | 1000 | 18.0 | 50 |
| Kallikrein 6 | 1000 | 26.0 | 50 |
| NGFbeta | 1000 | 26.4 | 50 |
| p53 | 1000 | 80.0 | 50 |
| PSA | 1000 | 53.0 | 50 |
| Pselectin | 1000 | 28.0 | 50 |
| TIMP-1 | 1000 | 21.0 | 50 |
| Timp4 | 1000 | 22.0 | 50 |
| TNFa | 1000 | 17.5 | 50 |
| VEGF | 1000 | 28.0 | 50 |

**Supplementary Table 1. Conversion of molar concentrations to concentrations in pg/μl.** The highest molar concentration (pM) for every protein used in the cross-reactivity experiment is converted to mass per volume concentration (pg/μl). In addition reaction volumes are provided.
