# Supplementary material for: ProteinSeq: High-Performance Proteomic Analyses by Proximity Ligation and Next Generation Sequencing
Source: PLoS One. 2011 Sep 29;6(9):e25583. doi: 10.1371/journal.pone.0025583 (PMC3183061; doi:10.1371/journal.pone.0025583)
Supplement: Table S2 — Detection limits for each protein included in the panel. The limits of detection for each protein included in the panel for both multiplex SP-PLA and sandwich immunoassays are presented in pg/ml. (DOCX) [file pone.0025583.s006.docx]

|  | **LOD (pM)** | | **LOD (pg/ml)** | |
| --- | --- | --- | --- | --- |
| **Protein name** | **Multiplex SP-PLA** | **Sandwich immunoassay** | **Multiplex SP-PLA** | **Sandwich immunoassay** |
| Artemin | 0.054 | 4.336 | 1.284 | 104.059 |
| Cathepsin B | 0.218 | 0.685 | 7.305 | 22.938 |
| Cathepsin S | 1.504 | 0.625 | 50.397 | 20.937 |
| CCL2 | 0.048 | 0.443 | 0.414 | 3.851 |
| CCL4 | 0.025 | 0.259 | 0.185 | 1.939 |
| CCL5 | 0.275 | 0.455 | 2.148 | 3.553 |
| CD40 ligand | 2.096 | 1.076 | 35.635 | 18.294 |
| CF3 | 0.361 | 0.584 | 9.382 | 15.186 |
| CXCL5 | 0.695 | 0.670 | 5.842 | 5.630 |
| Cystatin B | 0.693 | 0.570 | 8.317 | 6.841 |
| Cystatin C | 1.573 | 2.567 | 23.588 | 38.507 |
| E-selectin | 0.007 | 0.040 | 0.428 | 2.359 |
| EGF | 0.148 | 6.359 | 0.885 | 38.156 |
| Fas/TNFRSF6 | 0.004 | 0.326 | 0.383 | 29.337 |
| Follistatin | 0.229 | 0.578 | 7.091 | 17.932 |
| GDF-15 | 0.025 | 0.185 | 0.639 | 4.804 |
| HCC-4/CCL 16 | 0.231 | 0.591 | 2.540 | 6.499 |
| HGH | 0.155 | 0.889 | 3.416 | 19.556 |
| ICAM | 0.008 | 0.050 | 0.378 | 2.516 |
| IL-17A | 6.790 | 8.561 | 126.296 | 159.240 |
| IL1 alpha | 0.272 | 0.054 | 8.692 | 1.743 |
| IL10 | 1.641 | 49.580 | 22.971 | 694.117 |
| IL4 | 1.744 | 0.485 | 35.403 | 9.842 |
| IL6 | 0.072 | 0.095 | 1.228 | 1.609 |
| IL7 | 0.006 | 0.389 | 0.044 | 3.109 |
| IL8 | 0.017 | 0.201 | 0.302 | 3.627 |
| Kallikrein 6 | 1.643 | 0.833 | 42.713 | 21.671 |
| NGFbeta | 0.970 | 4.291 | 25.619 | 113.270 |
| p53 | 0.001 | 0.735 | 0.079 | 58.813 |
| PSA | 0.047 | 0.286 | 2.490 | 15.182 |
| Pselectin | 0.145 | 0.386 | 4.049 | 10.816 |
| TIMP-1 | 0.238 | 0.609 | 5.007 | 12.785 |
| Timp4 | 0.222 | 0.793 | 4.888 | 17.438 |
| TNFa | 2.992 | 0.452 | 52.359 | 7.903 |
| VEGF | 0.029 | 0.427 | 0.824 | 11.961 |

**Supplementary Table 2. Detection limits for each protein included in the panel.** The limits of detection for each protein included in the panel for both multiplex SP-PLA and sandwich immunoassays are presented in pg/ml.
