# Supplementary material for: ProteinSeq: High-Performance Proteomic Analyses by Proximity Ligation and Next Generation Sequencing
Source: PLoS One. 2011 Sep 29;6(9):e25583. doi: 10.1371/journal.pone.0025583 (PMC3183061; doi:10.1371/journal.pone.0025583)
Supplement: Table S3 — Adjusted concentration of each PLA probe. Probe concentrations were adjusted in order to decrease PLA reporting efficiency and thus limit the dynamic range of different ligation products. (DOCX) [file pone.0025583.s007.docx]

| **Protein** | **Proximity probe 1 (pM)** | **Proximity probe 2 (pM)** |
| --- | --- | --- |
| HCC-4 | 0.1 | 500 |
| TIMP-1 | 0.1 | 500 |
| Mouse IgG | 0.4 | 500 |
| ICAM-1 | 0.4 | 500 |
| CCL5 | 0.5 | 500 |
| Cystatin C | 0.6 | 500 |
| E-selectin | 2.0 | 500 |
| Cathepsin B | 3.6 | 500 |
| Timp4 | 3.6 | 500 |
| PSA | 7.1 | 500 |
| Pselectin | 7.1 | 500 |
| Kallikrein 6 | 20 | 500 |
| Cystatin B | 20 | 500 |
| Cathepsin S | 50 | 500 |
| IL8 | 50 | 500 |
| Fas | 50 | 500 |
| GDF-15 | 50 | 500 |
| VEGF | 500 | 500 |
| Follistatin | 500 | 500 |
| IL7 | 500 | 500 |
| CCL4 | 500 | 500 |
| IL10 | 500 | 500 |
| p53 | 500 | 500 |
| HGH | 500 | 500 |
| Artemin | 500 | 500 |
| CD40L | 500 | 500 |
| TNFa | 500 | 500 |
| CXCL5 | 500 | 500 |
| NGFbeta | 500 | 500 |
| IL6 | 500 | 500 |
| IL1 alpha | 500 | 500 |
| CCL2 | 500 | 500 |
| EGF | 500 | 500 |
| IL-17A | 500 | 500 |
| CF3 | 500 | 500 |
| IL4 | 500 | 500 |

**Supplementary Table 3. Adjusted concentration of each PLA probe.** Probe concentrations were adjusted in order to decrease PLA reporting efficiency and thus limit the dynamic range of different ligation products.
