# Supplementary material for: ProteinSeq: High-Performance Proteomic Analyses by Proximity Ligation and Next Generation Sequencing
Source: PLoS One. 2011 Sep 29;6(9):e25583. doi: 10.1371/journal.pone.0025583 (PMC3183061; doi:10.1371/journal.pone.0025583)
Supplement: Table S4 — List of antibodies used as PLA probes. (DOCX) [file pone.0025583.s008.docx]

| **Protein** | **Species** | **Type** | **Purification** | **Supplier** | **Modification** | **Catalog Number** |
| --- | --- | --- | --- | --- | --- | --- |
| Human Artemin | Goat | Polyclonal | Affinity purified | R&D Systems | None | AF-2589 |
| Human Artemin | Goat | Polyclonal | Affinity purified | R&D Systems | Biotin | BAF-2589 |
| Human beta-NGF | Goat | Polyclonal | Affinity purified | R&D Systems | None | AF-256 |
| Human beta-NGF | Goat | Polyclonal | Affinity purified | R&D Systems | Biotin | BAF-256 |
| Human Cathepsin-B | Goat | Polyclonal | Affinity purified | R&D Systems | None | AF-953 |
| Human Cathepsin-B | Goat | Polyclonal | Affinity purified | R&D Systems | Biotin | BAF-953 |
| Human Cathepsin-S | Goat | Polyclonal | Affinity purified | R&D Systems | None | AF-1183 |
| Human Cathepsin-S | Goat | Polyclonal | Affinity purified | R&D Systems | Biotin | BAF-1183 |
| Human CCL16/HCC-4 | Goat | Polyclonal | Affinity purified | R&D Systems | None | AF-802 |
| Human CCL16/HCC-4 | Goat | Polyclonal | Affinity purified | R&D Systems | Biotin | BAF-802 |
| Human CCL2/MCP-1 | Goat | Polyclonal | Affinity purified | R&D Systems | None | AF-279 |
| Human CCL2/MCP-1 | Goat | Polyclonal | Affinity purified | R&D Systems | Biotin | BAF-279 |
| Human CCL4/MIP-1 beta | Goat | Polyclonal | Affinity purified | R&D Systems | None | AF-271 |
| Human CCL4/MIP-1 beta | Goat | Polyclonal | Affinity purified | R&D Systems | Biotin | BAF-271 |
| Human CCL5/RANTES | Goat | Polyclonal | Affinity purified | R&D Systems | None | AF-278 |
| Human CCL5/RANTES | Goat | Polyclonal | Affinity purified | R&D Systems | Biotin | BAF-278 |
| Human CD40Ligand/TNFSF5 | Goat | Polyclonal | Affinity purified | R&D Systems | None | AF-617 |
| Human CD40Ligand/TNFSF5 | Goat | Polyclonal | Affinity purified | R&D Systems | Biotin | BAF-617 |
| Human Coagulation Factor III/Tissue Factor | Goat | Polyclonal | Affinity purified | R&D Systems | None | AF-2339 |
| Human Coagulation Factor III/Tissue Factor | Goat | Polyclonal | Affinity purified | R&D Systems | Biotin | BAF-2339 |
| Human CXCL5/ENA-78 | Goat | Polyclonal | Affinity purified | R&D Systems | None | AF-254 |
| Human CXCL5/ENA-78 | Goat | Polyclonal | Affinity purified | R&D Systems | Biotin | BAF-254 |
| Human CXCL8/IL-8 | Goat | Polyclonal | Affinity purified | R&D Systems | None | AF-208 |
| Human CXCL8/IL-8 | Goat | Polyclonal | Affinity purified | R&D Systems | Biotin | BAF-208 |
| Human Cystatin-B | Goat | Polyclonal | Affinity purified | R&D Systems | None | AF-1408 |
| Human Cystatin-B | Goat | Polyclonal | Affinity purified | R&D Systems | Biotin | BAF-1408 |
| Human Cystatin-C | Goat | Polyclonal | Affinity purified | R&D Systems | None | AF-1196 |
| Human Cystatin-C | Goat | Polyclonal | Affinity purified | R&D Systems | Biotin | BAF-1196 |
| Human E-selectin | Sheep | Polyclonal | Affinity purified | R&D Systems | None | AF-724 |
| Human E-selectin | Sheep | Polyclonal | Affinity purified | R&D Systems | Biotin | BAF-724 |
| Human EGF | Goat | Polyclonal | Affinity purified | R&D Systems | None | AF-236 |
| Human EGF | Goat | Polyclonal | Affinity purified | R&D Systems | Biotin | BAF-236 |
| Human Fas/TNFRSF6 | Goat | Polyclonal | Affinity purified | R&D Systems | None | AF-326 |
| Human Fas/TNFRSF6 | Goat | Polyclonal | Affinity purified | R&D Systems | Biotin | BAF-326 |
| Human Follistatin | Goat | Polyclonal | Affinity purified | R&D Systems | None | AF-669 |
| Human Follistatin | Goat | Polyclonal | Affinity purified | R&D Systems | Biotin | BAF-669 |
| Human GDF-15 | Goat | Polyclonal | Affinity purified | R&D Systems | None | AF-957 |
| Human GDF-15 | Goat | Polyclonal | Affinity purified | R&D Systems | Biotin | BAF-957 |
| Human Growth Hormone | Goat | Polyclonal | Affinity purified | R&D Systems | None | AF-1067 |
| Human Growth Hormone | Goat | Polyclonal | Affinity purified | R&D Systems | Biotin | BAF-1067 |
| Human ICAM-1/CD54 | Sheep | Polyclonal | Affinity purified | R&D Systems | None | AF-720 |
| Human ICAM-1/CD54 | Sheep | Polyclonal | Affinity purified | R&D Systems | Biotin | BAF-720 |
| Human IL-1 alpha/IL-1F1 | Goat | Polyclonal | Affinity purified | R&D Systems | None | AF-200 |
| Human IL-1 alpha/IL-1F1 | Goat | Polyclonal | Affinity purified | R&D Systems | Biotin | BAF-200 |
| Human IL-10 | Goat | Polyclonal | Affinity purified | R&D Systems | None | AF-217 |
| Human IL-10 | Goat | Polyclonal | Affinity purified | R&D Systems | Biotin | BAF-217 |
| Human IL-17 | Goat | Polyclonal | Affinity purified | R&D Systems | None | AF-317 |
| Human IL-17 | Goat | Polyclonal | Affinity purified | R&D Systems | Biotin | BAF-317 |
| Human IL-4 | Goat | Polyclonal | Affinity purified | R&D Systems | None | AF-204 |
| Human IL-4 | Goat | Polyclonal | Affinity purified | R&D Systems | Biotin | BAF-204 |
| Human IL-6 | Goat | Polyclonal | Affinity purified | R&D Systems | None | AF-206 |
| Human IL-6 | Goat | Polyclonal | Affinity purified | R&D Systems | Biotin | BAF-206 |
| Human IL-7 | Goat | Polyclonal | Affinity purified | R&D Systems | None | AF-207 |
| Human IL-7 | Goat | Polyclonal | Affinity purified | R&D Systems | Biotin | BAF-207 |
| Human Kallikrein-3/PSA | Goat | Polyclonal | Affinity purified | R&D Systems | None | AF-1344 |
| Human Kallikrein-3/PSA | Goat | Polyclonal | Affinity purified | R&D Systems | Biotin | BAF-1344 |
| Human Kallkrein-6/Neurosin | Goat | Polyclonal | Affinity purified | R&D Systems | None | AF-2008 |
| Human Kallkrein-6/Neurosin | Goat | Polyclonal | Affinity purified | R&D Systems | Biotin | BAF-2008 |
| Human P-Selectin/CD62P | Sheep | Polyclonal | Affinity purified | R&D Systems | None | AF-137 |
| Human P-Selectin/CD62P | Sheep | Polyclonal | Affinity purified | R&D Systems | Biotin | BAF-137 |
| Human p53 | Goat | Polyclonal | Affinity purified | R&D Systems | None | AF-1355 |
| Human p53 | Goat | Polyclonal | Affinity purified | R&D Systems | Biotin | BAF-1355 |
| Human TIMP-1 | Goat | Polyclonal | Affinity purified | R&D Systems | None | AF-970 |
| Human TIMP-1 | Goat | Polyclonal | Affinity purified | R&D Systems | Biotin | BAF-970 |
| Human TIMP-4 | Goat | Polyclonal | Affinity purified | R&D Systems | None | AF-974 |
| Human TIMP-4 | Goat | Polyclonal | Affinity purified | R&D Systems | Biotin | BAF-974 |
| Human TNF-alpha | Goat | Polyclonal | Affinity purified | R&D Systems | None | AF-210 |
| Human TNF-alpha | Goat | Polyclonal | Affinity purified | R&D Systems | Biotin | BAF-210 |
| Human VEGF 165 | Goat | Polyclonal | Affinity purified | R&D Systems | None | AF-293 |
| Human VEGF 165 | Goat | Polyclonal | Affinity purified | R&D Systems | Biotin | BAF-293 |
| Mouse IgG | Goat | Polyclonal | Affinity purified | R&D Systems | None | AF-007 |
| Mouse IgG | Goat | Polyclonal | Affinity purified | R&D Systems | Biotin | BAF-007 |

**Supplementary Table 4. List of antibodies used as PLA probes.**
