# Supplementary material for: ProteinSeq: High-Performance Proteomic Analyses by Proximity Ligation and Next Generation Sequencing
Source: PLoS One. 2011 Sep 29;6(9):e25583. doi: 10.1371/journal.pone.0025583 (PMC3183061; doi:10.1371/journal.pone.0025583)
Supplement: Table S5 — Sequences of PLA arms. Sequences of all oligonucleotides conjugated on antibodies by their 5′, which was modified by addition of a thiol group. (DOCX) [file pone.0025583.s009.docx]

| **Name** | **Sequence of oligonucleotides with free 3’ ends** |
| --- | --- |
| Probe 1 | AAAAACGATTCGAGAACGTGACTGCCATGGCTATTATGATGTCTGAGGCCCTATCCCTG |
| Probe 3 | AAAAACGATTCGAGAACGTGACTGCCATGTACCTCTATTGATACGTGGGTATCCCTGAG |
| Probe 5 | AAAAACGATTCGAGAACGTGACTGCCATGAATAGAATCCCTACGCCTAGCCCTGAGTCT |
| Probe 6 | AAAAACGATTCGAGAACGTGACTGCCATGCTTTCAAGTACCTTAGCTCGCCTGAGTCTA |
| Probe 9 | AAAAACGATTCGAGAACGTGACTGCCATGTAGTCAGGTTGGATGTCTACATCCCTGAGT |
| Probe 12 | AAAAACGATTCGAGAACGTGACTGCCATGAAGGTAATCTACACTACGGGGTCTATATGA |
| Probe 14 | AAAAACGATTCGAGAACGTGACTGCCATGCAATCATATCTAACCGGCTGCTATATGATG |
| Probe 15 | AAAAACGATTCGAGAACGTGACTGCCATGACTATCCGTCACTCAAGTAGTATATGATGA |
| Probe 16 | AAAAACGATTCGAGAACGTGACTGCCATGCCGATACTTAAAGCGTAGTGATATGATGAC |
| Probe 17 | AAAAACGATTCGAGAACGTGACTGCCATGGCTTACTATTCATACTGCCGTATGATGACT |
| Probe 18 | AAAAACGATTCGAGAACGTGACTGCCATGCCTATCGCTCTATATCTGGGATGATGACTA |
| Probe 20 | AAAAACGATTCGAGAACGTGACTGCCATGCGCTCTTATACCACTGTAGAGATGACTATC |
| Probe 22 | AAAAACGATTCGAGAACGTGACTGCCATGCGCGTATCTCTCTCTAGTAGTGACTATCGT |
| Probe 23 | AAAAACGATTCGAGAACGTGACTGCCATGGCAGTCTATTCCACTCAATGGACTATCGTA |
| Probe 24 | AAAAACGATTCGAGAACGTGACTGCCATGGTAACTGCTTCGTATCAAGGACTATCGTAC |
| Probe 25 | AAAAACGATTCGAGAACGTGACTGCCATGCGTCATCATTACTCACAGTGCTATCGTACC |
| Probe 28 | AAAAACGATTCGAGAACGTGACTGCCATGGCGTGTGATTCCTAGTAATGCGTACCCTGC |
| Probe 29 | AAAAACGATTCGAGAACGTGACTGCCATGCCAGCTCGTACTATCGAATAATCGTACCCT |
| Probe 30 | AAAAACGATTCGAGAACGTGACTGCCATGCCGCACTCTCGTAATATAGAGTACCCTGCA |
| Probe 31 | AAAAACGATTCGAGAACGTGACTGCCATGTATTCTAGTTCTGGACACGGTACCCTGCAA |
| Probe 33 | AAAAACGATTCGAGAACGTGACTGCCATGGTACTTCTGAAACGTGATGGCCCTGCAACT |
| Probe 34 | AAAAACGATTCGAGAACGTGACTGCCATGCCATAGTATCCTGTAAGCGTCCTGCAACTG |
| Probe 35 | AAAAACGATTCGAGAACGTGACTGCCATGACTGCTCAATCCTAGATACGCTGCAACTGT |
| Probe 37 | AAAAACGATTCGAGAACGTGACTGCCATGAATAGACATCAGTAGCTCCGGCAACTGTCG |
| Probe 38 | AAAAACGATTCGAGAACGTGACTGCCATGGTGACAGATTCTATGTGTGCCAACTGTCGC |
| Probe 40 | AAAAACGATTCGAGAACGTGACTGCCATGCGGTCCTCTGCATCTATAATACTGTCGCAT |
| Probe 42 | AAAAACGATTCGAGAACGTGACTGCCATGCCGCTGCATCTACTATAAGATGTCGCATCT |
| Probe 43 | AAAAACGATTCGAGAACGTGACTGCCATGGCTCTAATGTTAAGTGCTCGGTCGCATCTG |
| Probe 45 | AAAAACGATTCGAGAACGTGACTGCCATGGTCGCATATCGTTCTACCTACGCATCTGTA |
| Probe 46 | AAAAACGATTCGAGAACGTGACTGCCATGGGCGCATATCTGTCTATACTGCATCTGTAG |
| Probe 47 | AAAAACGATTCGAGAACGTGACTGCCATGACTCGTATATCATAAGCCCGCATCTGTAGC |
| Probe 48 | AAAAACGATTCGAGAACGTGACTGCCATGCAACTAAGTGCTACCGTCTAATCTGTAGCA |
| Probe 50 | AAAAACGATTCGAGAACGTGACTGCCATGCTGATGCTTAACTCGTATGCCTGTAGCAGT |
| Probe 53 | AAAAACGATTCGAGAACGTGACTGCCATGCTCCTAGCTTATGACAGCATTAGCAGTCTG |
| Probe 56 | AAAAACGATTCGAGAACGTGACTGCCATGTACAGCTATTGGAGACGATGCAGTCTGCCG |
| Probe 58 | AAAAACGATTCGAGAACGTGACTGCCATGATGTATCCGAAGTCGTAGTGGTCTGCCGCG |

**Supplementary Table 5. Sequences of PLA arms.** Sequences of all oligonucleotides conjugated on antibodies by their 5’, which was modified by addition of a thiol group.
