# Supplementary material for: ProteinSeq: High-Performance Proteomic Analyses by Proximity Ligation and Next Generation Sequencing
Source: PLoS One. 2011 Sep 29;6(9):e25583. doi: 10.1371/journal.pone.0025583 (PMC3183061; doi:10.1371/journal.pone.0025583)
Supplement: Table S6 — Sequences of PLA arms. Sequences of all oligonucleotides conjugated on antibodies by their 3′, which was modified by addition of a thiol group. (DOCX) [file pone.0025583.s010.docx]

| **Name** | **Sequence of oligonucleotides with free 5’ ends** |
| --- | --- |
| Probe 1 | AGTCTATATGCCCGATGCTGAATTAGTATCGATCGATACCGGACCAGGTTTCGCAAAAA |
| Probe 3 | TCTATATGATGACCTCACATTGACCATAAGGATCGATACCGGACCAGGTTTCGCAAAAA |
| Probe 5 | ATATGATGACGAGCTGAGTGAATCTTTCTCGATCGATACCGGACCAGGTTTCGCAAAAA |
| Probe 6 | TATGATGACTCCCGTTAGACTATCTGCTATGATCGATACCGGACCAGGTTTCGCAAAAA |
| Probe 9 | CTATATGATGTAGATACCGATACAGTGCTCGATCGATACCGGACCAGGTTTCGCAAAAA |
| Probe 12 | TGACTATCGTCACATACGCTACAGTTACAGGATCGATACCGGACCAGGTTTCGCAAAAA |
| Probe 14 | ACTATCGTACGCCCGTATGGTAACATTATCGATCGATACCGGACCAGGTTTCGCAAAAA |
| Probe 15 | CTATCGTACCCAGTAAGTATAACGAGCTGCGATCGATACCGGACCAGGTTTCGCAAAAA |
| Probe 16 | TATCGTACCCCACCGACGCTAATAGTTAAGGATCGATACCGGACCAGGTTTCGCAAAAA |
| Probe 17 | ATCGTACCCTGCGACGTATAGATTCGTATGGATCGATACCGGACCAGGTTTCGCAAAAA |
| Probe 18 | TCGTACCCTGTAGCACTGGACATAGATGAGGATCGATACCGGACCAGGTTTCGCAAAAA |
| Probe 20 | GTACCCTGCACGGTACTCAGTCATAGTGATGATCGATACCGGACCAGGTTTCGCAAAAA |
| Probe 22 | ACCCTGCAACGAGATGACGTGACATATTCGGATCGATACCGGACCAGGTTTCGCAAAAA |
| Probe 23 | CCCTGCAACTCCCGCATTATTATACACGAGGATCGATACCGGACCAGGTTTCGCAAAAA |
| Probe 24 | CCTGCAACTGGCCCGTTCTCTACAGTATATGATCGATACCGGACCAGGTTTCGCAAAAA |
| Probe 25 | CTGCAACTGTCAGACGACTATCGCATAATGGATCGATACCGGACCAGGTTTCGCAAAAA |
| Probe 28 | AACTGTCGCAGCTCCTCGCTAATAAGAAACGATCGATACCGGACCAGGTTTCGCAAAAA |
| Probe 29 | GCAACTGTCGCAGGTGTATAGAGTTCTGCTGATCGATACCGGACCAGGTTTCGCAAAAA |
| Probe 30 | ACTGTCGCATCCGGTTATCCAAGTGTACTAGATCGATACCGGACCAGGTTTCGCAAAAA |
| Probe 31 | CTGTCGCATCCATACTCTTGAAGCGATGTCGATCGATACCGGACCAGGTTTCGCAAAAA |
| Probe 33 | GTCGCATCTGCACGGTATTTAATCGGATGGGATCGATACCGGACCAGGTTTCGCAAAAA |
| Probe 34 | TCGCATCTGTCTACATAGACGATAGGGACGGATCGATACCGGACCAGGTTTCGCAAAAA |
| Probe 35 | CGCATCTGTAACACTCACGACATATAGGTCGATCGATACCGGACCAGGTTTCGCAAAAA |
| Probe 37 | CATCTGTAGCGCCTAGTCACCAATATACGAGATCGATACCGGACCAGGTTTCGCAAAAA |
| Probe 38 | ATCTGTAGCACTTCATATAGTGAGAGGCGTGATCGATACCGGACCAGGTTTCGCAAAAA |
| Probe 40 | CTGTAGCAGTCTCAGCCGAGTATATTGACTGATCGATACCGGACCAGGTTTCGCAAAAA |
| Probe 42 | GTAGCAGTCTCATTTGCAGTAAGTCGGTAGGATCGATACCGGACCAGGTTTCGCAAAAA |
| Probe 43 | TAGCAGTCTGCACGAGTGCTAAGATTTGAGGATCGATACCGGACCAGGTTTCGCAAAAA |
| Probe 45 | GCAGTCTGCCAGGCAGATACGATTAGTACGGATCGATACCGGACCAGGTTTCGCAAAAA |
| Probe 46 | CAGTCTGCCGCGACGATGCTTATAGACTCTGATCGATACCGGACCAGGTTTCGCAAAAA |
| Probe 47 | AGTCTGCCGCCAACGGATAGTTAGTCAGGTGATCGATACCGGACCAGGTTTCGCAAAAA |
| Probe 48 | GTCTGCCGCGGACTGCGTATCATATAGGCTGATCGATACCGGACCAGGTTTCGCAAAAA |
| Probe 50 | CTGCCGCGCTGATACTACGACATCTAGGGCGATCGATACCGGACCAGGTTTCGCAAAAA |
| Probe 53 | CCGCGCTTTCACTATCTAACGAGCTTGGTCGATCGATACCGGACCAGGTTTCGCAAAAA |
| Probe 56 | CGCTTTCGCACCCGTGCATCAATAGTAGTAGATCGATACCGGACCAGGTTTCGCAAAAA |
| Probe 58 | CTTTCGCAGTGCTGGATGAGTACGTCTATTGATCGATACCGGACCAGGTTTCGCAAAAA |

**Supplementary Table 6. Sequences of PLA arms.** Sequences of all oligonucleotides conjugated on antibodies by their 3’, which was modified by addition of a thiol group.
