# Supplementary material for: ProteinSeq: High-Performance Proteomic Analyses by Proximity Ligation and Next Generation Sequencing
Source: PLoS One. 2011 Sep 29;6(9):e25583. doi: 10.1371/journal.pone.0025583 (PMC3183061; doi:10.1371/journal.pone.0025583)
Supplement: Table S7 — PCR primer sequences. Sequences of all PCR primers used in multiplex SP-PLA with qPCR readout. (DOCX) [file pone.0025583.s011.docx]

| **Name** | **Forward** | **Reverse** |
| --- | --- | --- |
| Universal primer | CGATTCGAGAACGTGACTGC | GCGAAACCTGGTCCGGTATC |
| Specific primer 1 | GCTATTATGATGTCTGAGGC | GATACTAATTCAGCATCGGG |
| Specific primer 3 | TACCTCTATTGATACGTGGG | CTTATGGTCAATGTGAGGTC |
| Specific primer 5 | AATAGAATCCCTACGCCTAG | GAGAAAGATTCACTCAGCTC |
| Specific primer 6 | CTTTCAAGTACCTTAGCTCG | ATAGCAGATAGTCTAACGGG |
| Specific primer 9 | TAGTCAGGTTGGATGTCTAC | GAGCACTGTATCGGTATCTA |
| Specific primer 12 | AAGGTAATCTACACTACGGG | CTGTAACTGTAGCGTATGTG |
| Specific primer 14 | CAATCATATCTAACCGGCTG | GATAATGTTACCATACGGGC |
| Specific primer 15 | ACTATCCGTCACTCAAGTAG | GCAGCTCGTTATACTTACTG |
| Specific primer 16 | CCGATACTTAAAGCGTAGTG | CTTAACTATTAGCGTCGGTG |
| Specific primer 17 | GCTTACTATTCATACTGCCG | CATACGAATCTATACGTCGC |
| Specific primer 18 | CCTATCGCTCTATATCTGGG | CTCATCTATGTCCAGTGCTA |
| Specific primer 20 | CGCTCTTATACCACTGTAGA | ATCACTATGACTGAGTACCG |
| Specific primer 22 | CGCGTATCTCTCTCTAGTAG | CGAATATGTCACGTCATCTC |
| Specific primer 23 | GCAGTCTATTCCACTCAATG | CTCGTGTATAATAATGCGGG |
| Specific primer 24 | GTAACTGCTTCGTATCAAGG | ATATACTGTAGAGAACGGGC |
| Specific primer 25 | CGTCATCATTACTCACAGTG | CATTATGCGATAGTCGTCTG |
| Specific primer 28 | GCGTGTGATTCCTAGTAATG | GTTTCTTATTAGCGAGGAGC |
| Specific primer 29 | CCAGCTCGTACTATCGAATA | AGCAGAACTCTATACACCTG |
| Specific primer 30 | CCGCACTCTCGTAATATAGA | TAGTACACTTGGATAACCGG |
| Specific primer 31 | TATTCTAGTTCTGGACACGG | GACATCGCTTCAAGAGTATG |
| Specific primer 33 | GTACTTCTGAAACGTGATGG | CCATCCGATTAAATACCGTG |
| Specific primer 34 | CCATAGTATCCTGTAAGCGT | CGTCCCTATCGTCTATGTAG |
| Specific primer 35 | ACTGCTCAATCCTAGATACG | GACCTATATGTCGTGAGTGT |
| Specific primer 37 | AATAGACATCAGTAGCTCCG | TCGTATATTGGTGACTAGGC |
| Specific primer 38 | GTGACAGATTCTATGTGTGC | ACGCCTCTCACTATATGAAG |
| Specific primer 40 | CGGTCCTCTGCATCTATAAT | AGTCAATATACTCGGCTGAG |
| Specific primer 42 | CCGCTGCATCTACTATAAGA | CTACCGACTTACTGCAAATG |
| Specific primer 43 | GCTCTAATGTTAAGTGCTCG | CTCAAATCTTAGCACTCGTG |
| Specific primer 45 | GTCGCATATCGTTCTACCTA | CGTACTAATCGTATCTGCCT |
| Specific primer 46 | GGCGCATATCTGTCTATACT | AGAGTCTATAAGCATCGTCG |
| Specific primer 47 | ACTCGTATATCATAAGCCCG | ACCTGACTAACTATCCGTTG |
| Specific primer 48 | CAACTAAGTGCTACCGTCTA | AGCCTATATGATACGCAGTC |
| Specific primer 50 | CTGATGCTTAACTCGTATGC | GCCCTAGATGTCGTAGTATC |
| Specific primer 53 | CTCCTAGCTTATGACAGCAT | GACCAAGCTCGTTAGATAGT |
| Specific primer 56 | TACAGCTATTGGAGACGATG | TACTACTATTGATGCACGGG |
| Specific primer 58 | ATGTATCCGAAGTCGTAGTG | AATAGACGTACTCATCCAGC |

**Supplementary Table 7. PCR primer sequences.** Sequences of all PCR primers used in multiplex SP-PLA with qPCR readout.
