# Supplementary material for: ProteinSeq: High-Performance Proteomic Analyses by Proximity Ligation and Next Generation Sequencing
Source: PLoS One. 2011 Sep 29;6(9):e25583. doi: 10.1371/journal.pone.0025583 (PMC3183061; doi:10.1371/journal.pone.0025583)
Supplement: Table S8 — Library preparation oligonucleotide sequences. Sequences of all oligonucleotides used for the preparation of sequencing libraries. (DOCX) [file pone.0025583.s012.docx]

| **Name** | **Sequence** |
| --- | --- |
| PCR Index 1 forward primer | AATGATACGGCGACCACCGAGATCTACACTCTTTCCCTACACGACGCTCTTCCGATCCGTGATGCGAAACCTGGTCCGGTATC |
| PCR Index 2 forward primer | AATGATACGGCGACCACCGAGATCTACACTCTTTCCCTACACGACGCTCTTCCGATCACATCGGCGAAACCTGGTCCGGTATC |
| PCR Index 3 forward primer | AATGATACGGCGACCACCGAGATCTACACTCTTTCCCTACACGACGCTCTTCCGATCGCCTAAGCGAAACCTGGTCCGGTATC |
| PCR Index 4 forward primer | AATGATACGGCGACCACCGAGATCTACACTCTTTCCCTACACGACGCTCTTCCGATCTGGTCAGCGAAACCTGGTCCGGTATC |
| PCR Index 5 forward primer | AATGATACGGCGACCACCGAGATCTACACTCTTTCCCTACACGACGCTCTTCCGATCCACTGTGCGAAACCTGGTCCGGTATC |
| PCR Index 6 forward primer | AATGATACGGCGACCACCGAGATCTACACTCTTTCCCTACACGACGCTCTTCCGATCATTGGCGCGAAACCTGGTCCGGTATC |
| PCR Index 7 forward primer | AATGATACGGCGACCACCGAGATCTACACTCTTTCCCTACACGACGCTCTTCCGATCGATCTGGCGAAACCTGGTCCGGTATC |
| PCR Index 8 forward primer | AATGATACGGCGACCACCGAGATCTACACTCTTTCCCTACACGACGCTCTTCCGATCTCAAGTGCGAAACCTGGTCCGGTATC |
| PCR Index 9 forward primer | AATGATACGGCGACCACCGAGATCTACACTCTTTCCCTACACGACGCTCTTCCGATCCTGATCGCGAAACCTGGTCCGGTATC |
| PCR Index 10 forward primer | AATGATACGGCGACCACCGAGATCTACACTCTTTCCCTACACGACGCTCTTCCGATCAAGCTAGCGAAACCTGGTCCGGTATC |
| PCR Index 11 forward primer | AATGATACGGCGACCACCGAGATCTACACTCTTTCCCTACACGACGCTCTTCCGATCGTAGCCGCGAAACCTGGTCCGGTATC |
| PCR Index 12 forward primer | AATGATACGGCGACCACCGAGATCTACACTCTTTCCCTACACGACGCTCTTCCGATCTACAAGGCGAAACCTGGTCCGGTATC |
| PCR Index reverse primer | CAAGCAGAAGACGGCATACGAGATCGATTCGAGAACGTGACTGC |
| Enrich forward primer | CAAGCAGAAGACGGCATACGAGAT |
| Enrich reverse primer | AATGATACGGCGACCACCGAGATCT |

**Supplementary Table 8. Library preparation oligonucleotide sequences.** Sequences of all oligonucleotides used for the preparation of sequencing libraries.
