# Supplementary material for: ProteinSeq: High-Performance Proteomic Analyses by Proximity Ligation and Next Generation Sequencing
Source: PLoS One. 2011 Sep 29;6(9):e25583. doi: 10.1371/journal.pone.0025583 (PMC3183061; doi:10.1371/journal.pone.0025583)
Supplement: Table S9 — Characteristics of patient and control groups. (DOCX) [file pone.0025583.s013.docx]

|  |  |  | **Age** |  |  | **Sex** |  |
| --- | --- | --- | --- | --- | --- | --- | --- |
|  | n | Mean | Median | Max | Min | Female | Male |
| **Patients** | 63 | 68.90 | 71.00 | 85.00 | 36.00 | 33% | 67% |
| **Controls** | 19 | 63.74 | 64.00 | 75.00 | 50.00 | 37% | 63% |

**Supplementary Table 9. Characteristics of patient and control groups.**
