# Supplementary material for: ProteinSeq: High-Performance Proteomic Analyses by Proximity Ligation and Next Generation Sequencing
Source: PLoS One. 2011 Sep 29;6(9):e25583. doi: 10.1371/journal.pone.0025583 (PMC3183061; doi:10.1371/journal.pone.0025583)
Supplement: Material S1 — Supplementary Material. (DOCX) [file pone.0025583.s014.docx]

**Supplementary Material**

**Multivariate Data Analysis**

*Imputations:* No imputations were performed. Instead, two patient samples containing missing values were removed from the original set of 63 patient samples. This resulted in 61 patient samples and 19 control samples.

*Supervised learning:* For the supervised design of classifiers to discriminate between patient and controls two commonly employed algorithms and one novel method developed in-house were employed. One of the commonly employed methods was nearest shrunken centroid (NSC) classification([Tibshirani et al, 2002](#_ENREF_4)) as implemented by the **R package “pamr” (**<http://CRAN.R-project.org/package=pamr>)**.** The other commonly employed method was random forest classification (RF)([Breiman, 2001](#_ENREF_1)) as implemented by the R package “random forest” ([Liaw A, 2002](#_ENREF_2)) . The novel method consists of a two-step procedure. In the first step, an optimal linear transformation is designed according to Okada, T. *et. al.*([Okada & Tomita, 1985](#_ENREF_3)) that compresses each of the original protein profiles into three coordinates by means of a linear projection. The basic idea is to use the design examples to determine three ‘meta’ proteins (each being a weighted sum of the original protein levels) that are maximizing a measure of discriminatory potency. In other words, this transformation is selected to produce a three dimensional representation of each patient/control sample that separates the two classes as well as possible. In the second step, 3-nearest neighbor classification was performed, assigning each query example to the same class as the majority class among its 3 nearest neighbors in the three-dimensional meta-protein space designed in the first step.

*Performance estimation:* To obtain estimates of the expected performance of the three supervised classifier design procedures employed, conventional 5-fold cross validations were performed repeatedly 1000 times, each time using a different randomly permuted order of the examples. Thus, this resulted in design of 5000 different classifiers, each designed using 48 patient examples plus 15 control examples and then tested using an external set of test examples consisting of 13 patients and 4 controls. Thus stratified cross validation was performed which means that the patient and control examples were sampled independently to preserve the relative proportions of patients and controls in each design and test sets. The two types of errors for the novel projection method, false negative rate and false positive rate, were 4% and 19% respectively. The corresponding estimates for the two state-of-the-art classifiers were slightly worse in this particular case; The NSC classifier estimates were 11% for the false negative rate and 26% for the false positive. For the RF classifier the false negative rate estimate was 2% while the false positive rate estimate was 25%.

*Standardization:* For each of the 5000 different designs, the design examples were used to normalize (standardize) the different protein levels to the same scale. This was achieved by first calculating the mean value for the design examples and then express both design and test examples in terms of fold-changes relative to the mean. In other words, a protein level *x_i_* is replaced by *x_i_/m_i_* where m_i_ is the mean value of that protein across the design examples.

*No information leaks:* Notably, to avoid an information leak that would result in an optimistic performance estimate, the corresponding external test examples were also standardized by means of the mean values obtained from the design set in each iteration. In other words, no information about the external test examples were used to determine the mean values used for normalization, a commonly encountered pitfall using in-house as well as commercial programs.

Breiman L (2001) Random Forests. *Machine Learning* **45:** 5-32

Liaw A WM (2002) Classification and Regression by random Forest. *R News* **2(3):** 18-22

Okada T, Tomita S (1985) An optimal orthonormal system for discriminant analysis. *Pattern Recognition* **18:** 139-144

Tibshirani R, Hastie T, Narasimhan B, Chu G (2002) Diagnosis of multiple cancer types by shrunken centroids of gene expression. *Proc Natl Acad Sci U S A* **99:** 6567-6572
